# Importance of mobile genetic element immunity in numerically abundant *Trichodesmium* clades

**DOI:** 10.1038/s43705-023-00214-y

**Published:** 2023-02-23

**Authors:** Eric A. Webb, Noelle A. Held, Yiming Zhao, Elaina D. Graham, Asa E. Conover, Jake Semones, Michael D. Lee, Yuanyuan Feng, Fei-xue Fu, Mak A. Saito, David A. Hutchins

**Affiliations:** 1 Marine and Environmental Biology, Department of Biological Sciences, University of Southern California, Los Angeles, CA 90089 USA; 2 Department of Marine Chemistry and Geochemistry, Woods Hole Oceanographic Institution, Woods Hole, MA 02543 USA; 3 Department of Earth, Atmospheric, and Planetary Sciences, Massachusetts Institute of Technology, Cambridge, MA 02139 USA; 4 Blue Marble Space Institute of Science, NASA Ames Research Center, Mountain View, CA 94035 USA; 5 College of Marine and Environmental Sciences, Tianjin University of Science and Technology, Tianjin, 300457 China; 6 Present Address: Department of Environmental Systems Science, ETH, Zurich, Switzerland

**Keywords:** Water microbiology, Biogeochemistry

## Abstract

The colony-forming cyanobacteria *Trichodesmium* spp. are considered one of the most important nitrogen-fixing genera in the warm, low nutrient ocean. Despite this central biogeochemical role, many questions about their evolution, physiology, and trophic interactions remain unanswered. To address these questions, we describe *Trichodesmium* pangenomic potential via significantly improved genomic assemblies from two isolates and 15 new >50% complete *Trichodesmium* metagenome-assembled genomes from hand-picked, *Trichodesmium* colonies spanning the Atlantic Ocean. Phylogenomics identified ~four N_2_ fixing clades of *Trichodesmium* across the transect, with *T. thiebautii* dominating the colony-specific reads. Pangenomic analyses showed that all *T. thiebautii* MAGs are enriched in COG defense mechanisms and encode a vertically inherited Type III-B Clustered Regularly Interspaced Short Palindromic Repeats and associated protein-based immunity system (CRISPR-Cas). Surprisingly, this CRISPR-Cas system was absent in all *T. erythraeum* genomes, vertically inherited by *T. thiebautii*, and correlated with increased signatures of horizontal gene transfer. Additionally, the system was expressed in metaproteomic and transcriptomic datasets and CRISPR spacer sequences with 100% identical hits to field-assembled, putative phage genome fragments were identified. While the currently CO_2_-limited *T. erythraeum* is expected to be a ‘winner’ of anthropogenic climate change, their genomic dearth of known phage resistance mechanisms, compared to *T. thiebautii*, could put this outcome in question. Thus, the clear demarcation of *T. thiebautii* maintaining CRISPR-Cas systems, while *T. erythraeum* does not, identifies *Trichodesmium* as an ecologically important CRISPR-Cas model system, and highlights the need for more research on phage-*Trichodesmium* interactions.

## Introduction

Low bioavailable concentrations of nitrogen can limit primary productivity in many oceanic euphotic zones (e.g. [1]). In the warm, oligotrophic open ocean, these low nitrogen concentrations select for nitrogen-fixing organisms that can efficiently convert atmospheric N_2_ to bioavailable forms [2]. While our understanding of nitrogen-fixing organisms in the oceans is evolving to include non-autotrophic diazotrophs and other unexpected physiologies (e.g. [3–6]), the filamentous, colony-forming cyanobacterium *Trichodesmium* is still considered a critical oceanic nitrogen fixer [3, 7].

Mariners have known filamentous *Trichodesmium spp*. as ‘sea-saw dust’ for hundreds of years because of the massive surface blooms they can form resembling small, water-suspended wood shavings [7]. *Trichodesmium* filaments can aggregate in natural communities, forming 1–4 mm colonies of essentially two morphologies (i.e., radial tufts or spherical puffs; [8]) that are visible to the naked eye and thus aided these early observations. Fitting with this long-term recognition, scientists defined six morphologically described species in the late 1800s [9]. Still, oceanographers did not recognize their central role in N_2_ fixation until the 1960s (e.g. [2, 3]). Researchers now know that *Trichodesmium* has a wide distribution in the tropics and subtropics [3, 10] and, even though some appear to have lost N_2_ fixation capabilities [4], the genus is still an essential source of bioavailable N to the oligotrophic oceans [7, 11]. Thus, while *Trichodesmium* species names have existed for >100 years, experiments to understand their evolution, genomic potential, and ecological impact are still active research areas.

Members of the *Trichodesmium* genus are closely related with large, repeat rich genomes [12, 13]. Yet, enrichment strains and field samples can show surprising morphological and physiological character variability (i.e., pigmentation, cell size, trichome shape, growth rate, N_2_ fixation rate, or colony structure) and abundance differences (e.g. [8, 14–16]). For example, marker gene phylogenetics shows four clades of *Trichodesmium* [8, 14, 17], with the best bootstrap support defining the *Trichodesmium thiebautii* and *Trichodesmium erythraeum*-enriched clades I and III, respectively [8]. Recent metagenomic work from the Red Sea has shown that some single gene metrics for *Trichodesmium* (i.e.*, hetR* gene) have led to misidentification of clades due to paralogous copies in certain genomes [13], but this work also supports the importance of the two major clades of *Trichodesmium*. Additionally, morphological and molecular fieldwork shows that members of these same two clades are commonly observed, although *T. thiebautii-*containing clade I is typically more abundant throughout the water column (e.g. [4, 10, 18, 19]). Thus, while there are six classically defined *Trichodesmium* species, *T. thiebautii* clade I typically dominates field populations. Despite this recognition, the internal and external factors causing the numerical dominance of *T. thiebautii* are poorly defined.

Herein we used metapangenomics and metaproteomics of enrichment cultures and hand-picked colonies spanning the Atlantic Ocean to define genomic features predicted to impact *Trichodesmium* population dynamics. Our efforts showed that predicted mobile genetic element immunity (i.e., against phage and mobile plasmids; MGE) is a defining feature of *T. thiebautii*, as all clade members maintain and express a conserved Type III-B CRISPR-Cas system [20].

## Materials and methods

### *Trichodesmium* colony collection

**Fig. 1:**
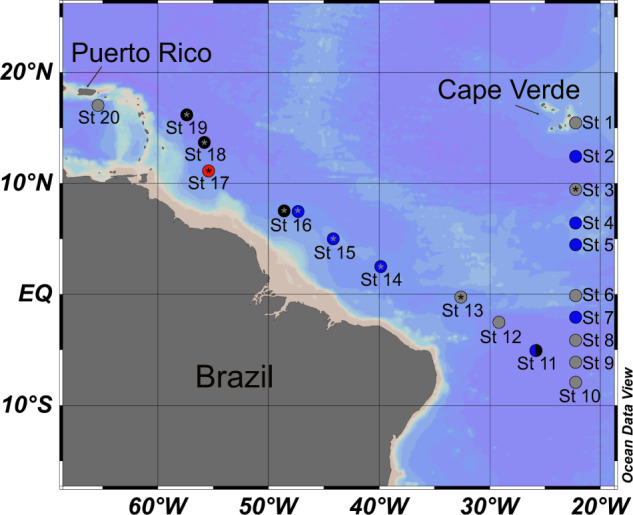
Map of the 2018 trans-Atlantic TriCoLim Cruise. Color of the station location indicates hand-picked *Trichodesmium* colony morphology, specifically puff (blue), tuft (black), combined (blue and black), not hand-picked (red), no metagenomic data (gray) and metaproteomic data (asterisk).

*Trichodesmium* colonies were collected with a hand-towed line (~150 ft) 130-µm Sea Gear plankton net on February 8th thru March 11th, 2018, during the R/V Atlantis TriCoLim cruise (AT39-05) that transected from Cape Verde to Puerto Rico (Fig. 1). Colonies were rapidly removed from the cod end and picked into tuft and puff morphologies with sterile plastic disposable Pasteur pipettes into 50 ml of sterilized seawater. These morphology-segregated samples were sequentially washed two times with sterile, local seawater, gently filtered down onto 5-µm polycarbonate membranes, and rapidly frozen in liquid N_2_.

### DNA isolation and sequencing

High-quality DNA was isolated from ~50 frozen colonies per station via Qiagen DNeasy Powersoil Kit (Germantown, MD) using the manufacturer’s protocol with the following exceptions. DNA quality and quantity was determined via NanoDrop UV-Vis spectrophotometer and Qubit Fluorometer, respectively (ThermoFisher; Waltham, MA) and twelve TriCoLim samples were 150 PE Illumina sequenced by Novogene (Sacramento, CA) to a final depth of 25 Gbps. DNA was isolated from frozen *T. thiebautii* H94 samples using the same protocol as above and was sequenced via 250PE Illumina MiSeq at the USC Epigenome Center (1.8 Gbps total) because the original assembly in [12] was poor quality. Raw reads are available at NCBI’s SRA under the BioProject PRJNA828267.

### Isolate and field MAG assembly

Both the field samples and *T. thiebautii* H94 reads were run through similar assembly pipelines, but H94 was assembled on KBase (https://www.kbase.us), while the former were on a Linux server. The quality of reads was checked with FastQC v0.11.2 [21] and trimmed to enhance stats using Trimmomatic v0.33 [22]. MAGs were assembled de novo using metaSPAdes v3.12.0 [23] for H94 and MEGAHIT v1.2.6 [24] for the TriCoLim samples. These programs were chosen because each yielded the best assembly stats. Binning of contigs was performed via MaxBin2 v2.2.4, quality was checked with CheckM v1.1.3 [25], and phylogenetic placement of the MAGs was determined with GTDB-tk v1.3.0 [26]. Field MAGs were dereplicated using fastANI [27] with a cutoff of 98.5% ID. Dereplicated bins >50% CheckM complete were hand refined in Anvi’o v7 [28, 29] until the contamination level was below 5%. *T. erythraeum* strain 2175 was downloaded from NCBI and hand-refined in Anvi’o [28, 29] to remove contaminating contigs using the TriCoLim reads, and its final genome stats were determined with CheckM [25].

### Phylogenomics

Higher quality *Trichodesmium* MAGs (>50% complete; Supplementary Table 1) and nearest relative genomes downloaded from the NCBI Assembly page were run through the program GToTree v1.6.12 to define a initial guide tree based on 251 cyanobacterial core protein Hidden Markov Models [30, 31]. Alignment and partition files from GToTree were piped to IQtree v2.1.4-beta in ModelFinder optimality mode (models LG + F + R10) with 1000 ultrafast bootstraps to generate the phylogenomic tree [32]. The tree was visualized and edited in the interactive Tree of Life (ITol; [33]).

### Metapangenomics

The metapangenomic pipeline in Anvi’o was used to characterize shared and distinct blast-defined gene clusters (GCs; mcl-inflation 10) in the MAGs and determine if these GCs were represented in the TriCoLim reads [28, 29]. Briefly, this pipeline creates a contig database for all MAGs that was then annotated with prodigal [34], COGs [35], PFAMS [36], KOFAM [37] and KEGG [38]. Reads were recruited to contigs in the Anvi’o database with TriCoLim read samples using bowtie2 v 2.4.1 [39], matching Sequence Alignment Map (SAMs) were converted to Binary Alignment Maps (BAMs) with SAMtools v1.11 [40], and BAMs were profiled across the TriCoLim read sets using Anvi’o to determine environmental auxiliary and environmental core genes (EAG and ECG, respectively). The reads were square root normalized to compress the results and allow one to visually see the presence of *T. erythraeum* in the recruitment heat map. COG categories per 100 kb was determined by exporting the annotation from Anvi’o, determining the COG categories per MAG, summing those results per clade, and then analyzing and graphing in R v 4.0.3 (2020-10-10) with R Studio v1.4.1103 [41]. Differences between COG category counts per clades were tested for statistical significance using ANOVA in the R package rstatix [42]. The same general pipeline was used to determine single gene copy per clade and toxin:antitoxin GCs per clade. The Anvi’o summary data was converted into a matrix via the scripts in [43] and used with the R package micropan [44] to generate Heaps’ law alpha value and genome fluidity estimates.

### CRISPR-Cas analyses

We scanned all *Trichodesmium* assemblies and those from their nearest relatives with CRISPRCasTyper [45]. This tool aids in the sometimes difficult task of identifying and typing CRISPR arrays and disparate *cas* loci based on the currently defined 44 subtypes and variants [20]. Because many of the MAGs were fragmented, CRISPR-Cas system portions on other contigs are shown with double slashes if (1) the pieces were found on the edges of their located contigs, and (2) the associated *cas* genes are still predicted to be part of the subtype III-B, I-D, or III-D systems defined in [20]. Additional annotations for accessory genes (purple) and hypothetical genes (gray) were determined by CRISPRCasFinder, and BLAST [46–48].

We used clustal in the program Geneious Prime (Biomatters, San Diego, CA) to align Cas10 protein sequences from all genomes, and RaxML v8 to generate the maximum likelihood phylogenetic tree with 100 bootstraps [49].

### Virome assembly

We screened for putative phage genome, prophage or plasmid fragments in TriCoLim and enrichment culture assemblies using the virsorter2, DRAM-V, and checkV pipelines for viruses and metaplasmidSPAdes for plasmids [50–53]. The contig sequences supplied from these efforts were used to generate custom blast databases [48] that were subsequently BLAST screened with *Trichodesmium* spacers defined above and grouped with FastANI [27].

### Proteome analysis of *Trichodesmium* enriched field samples

The raw proteome spectra were collected from *Trichodesmium* colony samples published in a prior work [54] and newly analyzed for this study. Specifically, raw spectra were searched with the Sequest algorithm implemented in Proteome Discoverer 2.2 using a custom-built *Trichodesmium* genomic database. To avoid inflation of the sequence database and later misinterpretation of phylogenetic signals, only one version of any identical/redundant protein sequences were included in the database, with the possible phylogenetic attributions for the redundant proteins noted in downstream phylogenetic analyses. Sequest mass tolerances were set to ±10ppm (parent) and ±0.6 Dalton (fragment). Fixed Cysteine modification of +57.022, and variable N-terminal acetylation of +42 and methionine modification of +16 were included. Protein identifications were made with Peptide Prophet in Scaffold (Proteome Software) at the 80% peptide threshold minimum, resulting in an estimated peptide false discovery rate (FDR) of 1.5% and an estimated protein FDR of 0.0%. Relative protein abundances are reported as normalized total exclusive spectral counts, so only spectra corresponding to a specific peptide for a given protein were considered. This avoids the problem of overlapping peptides in the phylogenetic analysis. The values are normalized to total spectral counts identified in each sample. The peptides identified for the CRISPR-Cas proteins were further checked using the Metatryp 2.0 tool (www.metatryp.whoi.edu) [55] to ensure phylogenetic specificity of the signals.

### Transcriptome read recruiting

*Trichodesmium* colony metatranscriptomes were downloaded from NCBI SRA (projects PRJNA381915 and PRJNA374879) and mapped against all *cas* genes from H94 and *cas10* from MAG *T. thiebautii* Indian Ocean that is a representative of cluster 3. Read quality was checked with FastQC v0.11.2 [21], trimmed with Trimmomatic v0.33 [22], recruited to cas genes with Bowtie v 2.4.1, converted to BAMs with Samtools v1.11, and profiled in Anvi’o v7.0. Average read depth values were normalized with the constitutive *Trichodesmium* gene *rotA*, as in [56]. The data were visualized in R v 4.0.3 (2020-10-10) with R Studio v1.4.1103.

## Results and discussion

### All N_2_-fixing*Trichodesmium* genomes are “low” protein-encoding

Past work with a handful of *Trichodesmium* isolates shows that their genomes are low protein-encoding (i.e., ~63%) and enriched in selfish DNA elements;[12] however, these observations have never been studied systematically across the genus. To address this, we assembled 15 new *Trichodesmium* MAGs from our hand-picked colonies obtained on the 2018 Atlantic Ocean spanning TriCoLim cruise (Fig. 1) and compared them to previously published isolate genomes [12] and three MAGs from a Tara Oceans analysis [4]. Two of our previously published USC *Trichodesmium* Culture Collection (USCTCC) genomes, *T. thiebautii* H94 and *T. erythraeum* 2175, were significantly improved by MiSeq resequencing the former and Anvi’o refining both using reads from TriCoLim. Supplementary Table 1 lists the final refined CheckM [25] statistics for all MAGs and genomes. Similar to a prior three isolate comparison [12], Supplementary Table 2 shows that higher-quality *Trichodesmium* MAGs have low GC% and much lower coding (~64%) than the bacterial average of ~90% [57], and are large, with an average length of ~6.5 ± 0.9 MB, and relatively gene sparse, only encoding for an average of ~5396 ± 784 proteins.

### There are four N_2_-fixing clades of *Trichodesmium*

**Fig. 2:**
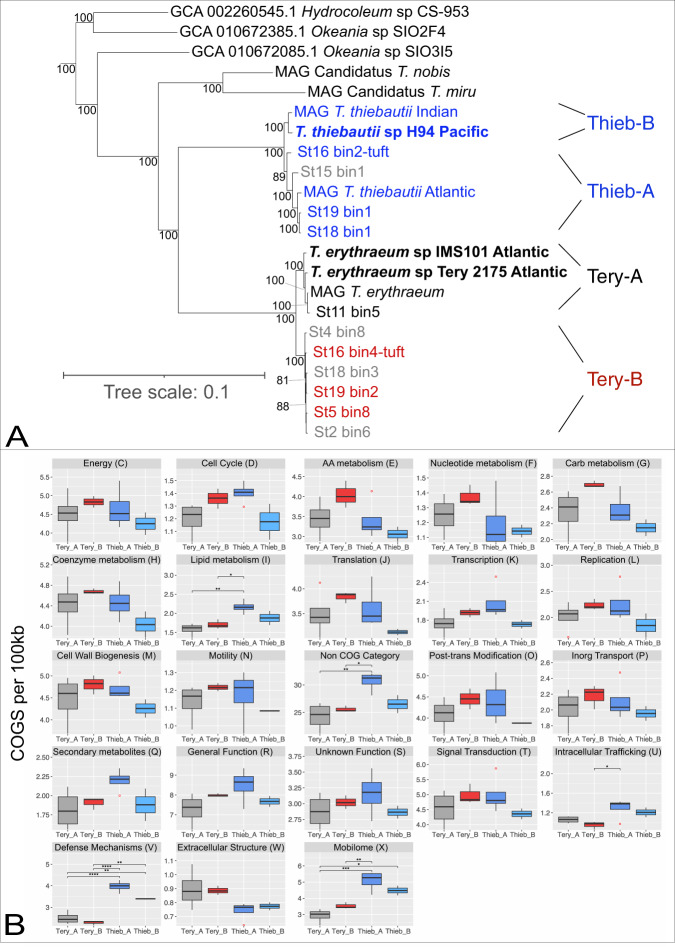
Phylogenomic tree of *Trichodesmium* and nearest relatives and COG enrichment per 100Kb of *Trichodesmium* clades. In (**A**), TriColim bins are named by station, USCTCC strains are in bold, and names beginning in “MAG” are from (4). *Trichodesmium* MAGs >80% complete are color coded by clade (i.e.)*, T. thiebautii* (Blue), *T. erythraeum* A (Black), *T. erythraeum* B (Red), while MAGs < 80% are shown in gray. Other *Trichodesmium* MAGs in Supplementary Table 1 were excluded from the tree due to low completion values. **B** shows the normalized and summed quantity of COG categories for each MAG per clade. Significantly different categories determined by ANOVA of the mean are denoted above the bracket (*p* < 0.05 = *, <0.01 = **, <0.001 = ***, <0.0001 = ****).

To improve *Trichodesmium* cladistics, we performed phylogenomics using 251 conserved core genes from *Trichodesmium* genomes and MAGs >50% complete. The resulting tree in Fig. 2A shows that the N_2_-fixing members of the genus are divided into four major clades and suggests that *T. thiebautii* assembled from the Atlantic (Clade A) are phylogenomically different from those from other basins (Clade B). However, our read recruiting and metaproteomics (see below) indicate that genomes with high identity to *T. thiebautii* B are also present and active in the Atlantic Ocean, but they did not assemble with high quality from TriCoLim samples (e.g., Supplementary Table 1; St11_bin2_1_1 and St14_bin2_1 are >98% ANI with *T. thiebautii* B H94 and MAG_*Trichodesmium_thiebautii*_Indian). Lastly, while Delmont has shown that a distinct branch of *Trichodesmium* are non-N_2_-fixers [4], BLAST searches of the TriCoLim MAGs with *T. erythraeum* IMS101 *nif* genes confirmed that all of these MAGs encode for diazotrophy (i.e., when missing in a MAG annotation, *nif* genes fragments were consistently found at the end of contigs).

Since there are no published isolate genomes for *T. thiebautii* clade A and *T. erythraeum* clade B, reconciling past species designations and predicting their physiological and morphological characters was not directly possible. However, we attempted to place these MAGs in context with previously isolated strains [8, 58] by comparing 16S-23S internal transcribed spacer (ITS) gene sequences via blast. The isolate ITS hits ranged from 86–100% ID to our MAGs and old strain/clade designations from Hynes et al (8) generally tracked well with our cladistics. However, the high identity hits (>99.5%) allowed us to make three conclusions. (1) *T. erythraeum* strains (6-1, 6-2, 6-5), that formed a weak subgroup in a prior analysis [8], are members of *T. erythraeum* clade B, and thus we predict this group has phycoerythrobilin-rich red cells ~6.5–9.5 µm in diameter that can form colonies or loose aggregates. (2) *Trichodesmium contortum* with larger diameter cells (~20–30 µm) and bright red coloration [8] is likely a member of *T. erythraeum* clade A. (3) The ITS does not contain enough information to determine if previous *Trichodesmium hildebrandtii, Trichodesmium tenue*, and *Trichodesmium spiralis* isolates are in either *T. thiebautii* clade A or B. Because of this taxonomic uncertainty, hereafter we forgo using other *Trichodesmium* species names and simply use the broad clade designations (i.e.*, T. erythraeum* A & B and *T. thiebautii* A & B).

### *Trichodesmium* genomes have many paralogous genes dominated by predicted mobile genetic elements

To understand broad-level genome evolution in the genus, we explored copy number enriched gene families in *Trichodesmium* genomes and MAGs. Our results show many paralogous GCs shared by all *Trichodesmium* genomes, with some found in very high copy numbers per clade. Interestingly, each clade’s top ten duplicated GCs are similar but not 100% identical in sequence or copy number (Supplementary Table 3). Thus, our data corroborate a prior finding that *Trichodesmium* genomes are repeat-rich [12] and show that these duplications are commonplace in situ. Annotation of these paralogous GCs shows that they are enriched (~78%) in “selfish DNA elements” like transposases, retrons, or group II introns. Since there is evidence that “selfish DNA elements” can be involved in bacterial genome re-arrangement or niche adaptation [59, 60], these results suggest that transposition or other related duplication generating processes may be important evolutionary mechanisms in *Trichodesmium*.

### *T. thiebautii* MAGs are enriched in specific clusters of orthologous genes (COG) compared to *T. erythraeum*

To begin to understand the selective pressures driving speciation in the genus, we next characterized the genomic potential of *Trichodesmium* in a phylogenomic context. At first glance, the average gene number per genome is greater in *T. thiebautii* than *T. erythraeum* (Supplementary Tables 2). As shown in Fig. 2B, many of the COG categories per 100 kb in each clade are statistically indistinguishable by ANOVA. However, there are five enriched categories in *T. thiebautii* A, *T. thiebautii* B, or both. These include Lipid Metabolism (I), Intracellular trafficking (U), Defense Mechanisms (V), Mobilome (X), and genes not categorized by COG. While most of these COG enrichments were dominated by transposases, repeat-filled genes, or hypothetical genes, the Lipid Metabolism and Defense genes had many informative annotations. Closer inspection of the Lipid Metabolism genes showed that *T. thiebautii* A has increased acyl-carrier proteins, many of which appear to be involved in polyketide synthases or annotated with multiple functions. These findings suggest increased secondary metabolite production in this clade. However, despite the interest in understanding how *Trichodesmium* acquires Fe (e.g. [16, 61],), none of these clusters are predicted to encode siderophores. Lastly, the *T. thiebautii* Defense category was enriched in putative toxin-antitoxin proteins [62] antiphage systems [63], and CRISPR-Cas genes [20], suggesting phage interactions are a selective pressure in the two *T. thiebautii* clades.

### One-quarter of all *Trichodesmium* MAGs have shared gene clusters

**Fig. 3:**
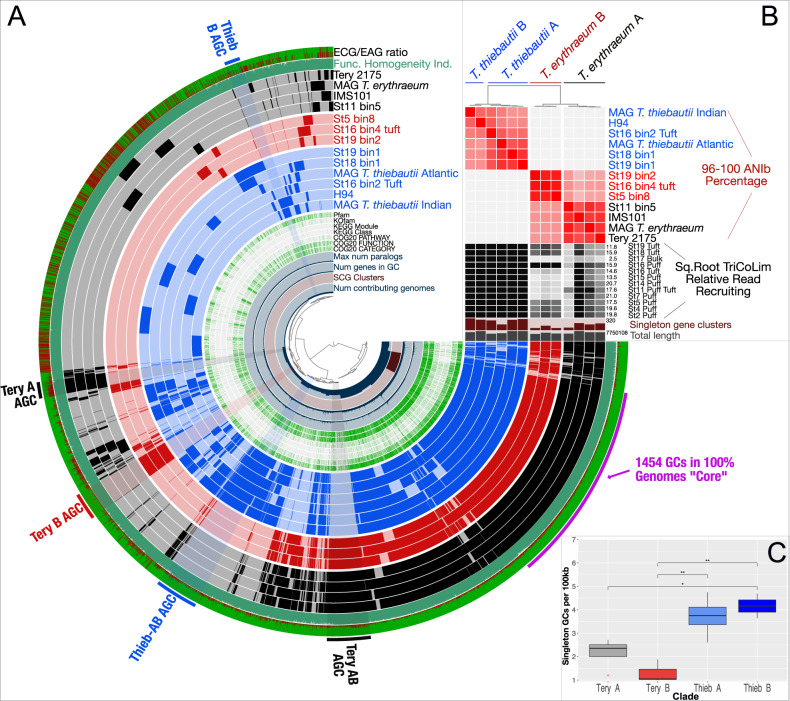
N_2_-fixing *Trichodesmium* Metapangenomic visualization. **A** shows blast-defined conserved gene clusters (GCs) in a MAG as filled colored rings (blue for all *T. thiebautii*, red for *T. erythraeum* B, and black for *T. erythraeum* A). Lighter fill colors indicate that those GCs are missing from that MAG. Singleton GCs (*i.e*., appearing in only one MAG) are mostly shown between 9 and 11 o’clock in the diagram. The innermost rings of the diagram indicate number of contributing genomes to a GC, single copy genes (SCG), number of genes in a GC, and max number of paralogs. Continuing outwards, if the GC has annotation it is marked in green, while if it does not it is white. The outermost two rings show whether a GC is environmentally core (green) or auxiliary (red; i.e., the redder the color, the less commonly the GC was observed in TriCoLim reads) and GC homogeneity (i.e., high homogeneity = all green fill). Clear groupings of clade specific auxiliary gene clusters (AGC) are labeled on the edge of the diagram. **B** shows BLAST ANI clustering at the top and the ANI heat map in red from 96 to 100. The black heat map shows square root normalized read recruiting to each MAG from TriCoLim (Blacker bars = higher relative read recruiting). **C** shows statistical analysis of singleton GCs per clade with ANOVA statistical support shown above the brackets (*p* < 0.05 = *, 0.01 = **).

To explore differences between *Trichodesmium* clades, we examined specific gene cluster presence/absence, annotated function, and detection in our TriCoLim reads. This effort allowed us to determine if the functionalities enriched or depleted in each clade in Fig. 2B were caused by distinct, new gene clusters, paralogous duplications, or deletions. Based on BLAST clustering, there are 1454 single and paralogous GCs in the conservative *Trichodesmium* core found in all genomes (Fig. 3A). Thus, approximately 1/4 of each genome is conserved core gene content. The total pangenome count was 10,054 genes. Pangenome modeling with the R package micropan [44] obtained Heap’s power law alpha estimates of ~1 for all *Trichodesmium* MAGs together and slightly >1 for *T. thiebautii* and *T. erythraeum* MAGs individually, indicating that these pangenomes are either “completely” sampled with this dataset (i.e., closed) or slowly growing logarithmically [64].

Others have argued that genome fluidity (ϕ), a metric of genome dissimilarity, is a better method for estimating the likelihood of identifying new genes as more genomes in a taxonomic group are sequenced [65, 66]. We determined the MAG genome fluidity values for all *Trichodesmium* (ϕ = 0.303 ± 0.10) and the major clades of *T. thiebautii* (ϕ = 0.24 ± 0.04), and *T. erythraeum* (ϕ = 0.18 ± 0.03). Strict interpretation of these data suggests a 30% chance of identifying new genes as more *Trichodesmium* genomes are sequenced—again fitting with a growing/open pangenome. Additionally, our data predicts that the likelihood of discovering new genes is higher as more *T. thiebautii* are sequenced compared to *T. erythraeum*. While it is important to note that these ϕ values will likely improve with increased numbers of genomes in each clade [65], the data are consistent with the *T. thiebautii* pangenome being more ‘open’ than *T. erythraeum* with the former likely experiencing increased horizontal gene transfer (HGT).

*T. thiebautii* dominated the read recruiting regardless of colony type, and intra-clade average nucleotide identity (ANI) of the MAGs was very high (Fig. 3B; black and red heat maps, respectively). Thus, in situ quantification of each MAG was not possible because of likely random read recruiting among high ANI genomes [67]. However, since this issue would likely only underestimate the abundances of each clade, we report that *T. thiebautii* MAGs were recruiting at least 1–2 orders of magnitude more reads than *T. erythraeum* from TriCoLim colonies.

### *Trichodesmium* auxiliary gene content and genomic average nucleotide identity (ANI) recapitulate the phylogenomic signal

While the predicted *Trichodesmium* core N_2_-fixing genome makes up ~1/4 of the genes, many auxiliary GCs are also detected. As shown in Fig. 3A, some auxiliary GCs were only found in one genome (i.e., singletons), while others associate with specific clades. The environmentally accessory genes (EAGs; i.e., not found in situ) to environmentally core genes (ECGs; i.e., found in situ) ratio shown on the outer ring indicates that many, but not all of these auxiliary GC bins, are commonly detected in Atlantic Ocean *Trichodesmium* colonies. Coloring the rings of Fig. 3A by phylogenomic group shows that the auxiliary gene content, average nucleotide identity (ANI; Fig. 3B), and phylogenomics of core genes (Fig. 2A) give the same relationships between *Trichodesmium* clades. Additionally, statistical analysis of singleton genes shows an uneven distribution in the genus, with *T. thiebautii* genomes maintaining significantly more (Fig. 3C). These empirical data are consistent with the genome fluidity results above and suggest mechanisms that increase novel gene content, like horizontal gene transfer, are more common in *T. thiebautii*.

We next took the GCs in each clade-specific bin, highlighted in Fig. 3A, to characterize enriched functionalities (Supplementary Fig. 1). The largest groups of clade-specific genes are found in the primary division between *T. thiebautii* AB and *T. erythraeum* AB, where the former shares 313 GCs and the latter shares 315, respectively. Percentage normalized COG analyses of these conserved GC bins showed four things: (1) non-COG categorized GCs dominate those found in all bins (ranging from ~44 to 79%), and 18–75% of these non-categorized GCs are hypotheticals, (2) the Tery-AB bin has much more COG diversity than the similarly sized Thieb-AB bin (Supplementary Fig. 1), (3) Thieb-AB, Thieb-B, and Tery-B bins are enriched in mobilome sequences (~10% of the bin’s GCs), while in Tery-A and Tery-AB, the mobilome GCs only account for ~5% of GCs, and (4) the Thieb-AB bin has a higher percentage of Defense COGs. While our data show that specific CRISPR-related gene duplications are common in *Trichodesmium* MAGs (Duplicated GCs; Supplementary Table 3), the Thieb-specific bin is enriched in CRISPR-Cas immunity genes.

### *T. thiebautii* encodes a complete Type III-B CRISPR-Cas system, while *T. erythraeum* does not

**Fig. 4:**
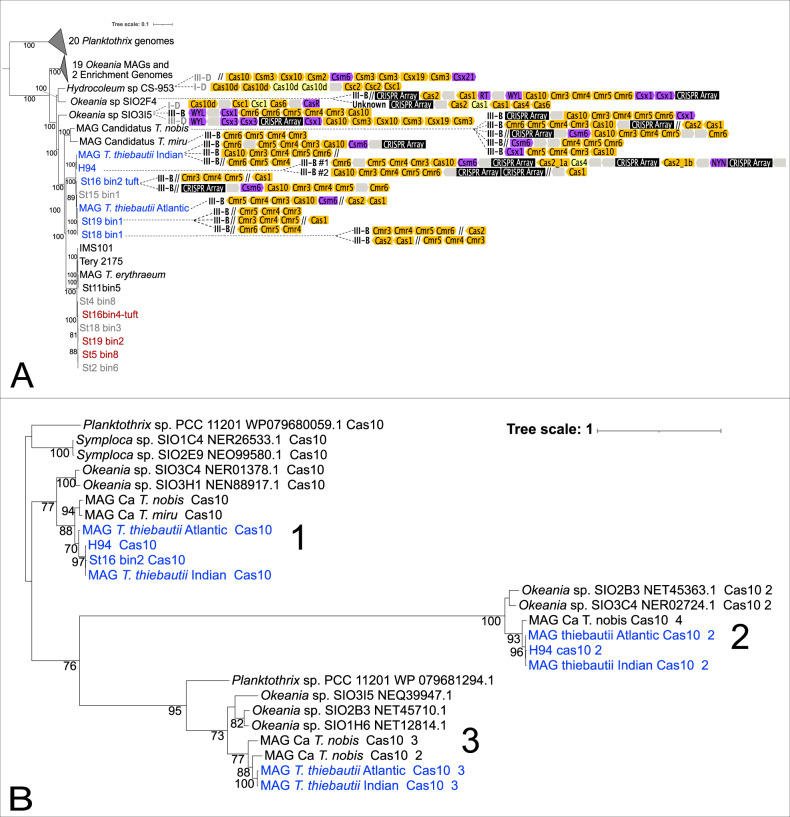
Presence of *Trichodesmium* CRISPR systems in a phylogenomic context and Cas10 phylogenetics. Presence or absence of CRISPR-Cas genes in a *Trichodesmium* MAGs and their nearest relatives phylogenomic context (**A**) and a maximum likelihood tree of their Cas10 protein sequences (**B**). In (**A**), the color-coded, directional shapes on the right represent detected Cas genes (yellow), CRISPR arrays (black), Cas accessory genes (purple) and hypothetical genes (gray) that were annotated by as described in the Materials and Methods. Lighter color indicates lower confidence in the annotation. Double-slashes are contig break positions near the annotated CRISPR-Cas systems, indicating that some clusters are fragmented due to breaks in the assemblies. Gene lengths are not drawn to scale. In (**B**), the color coding corresponds to *T. thiebautii* (Blue) and other relatives (Black). Cas10 protein sequences are in Supplementary Table 12.

The expanded, *Trichodesmium-*nearest relative phylogenomic tree in Fig. 4A shows *cas* gene detection in the lineage. These data demonstrate that the non-N_2_ fixing *Trichodesmium, T. thiebautii, Hydrocoleum* and *Okeania* MAGs all encode CRISPRs. In contrast, none of the 10 *T. erythraeum* MAGs analyzed here have them. Additionally, we could not identify any *cas10* gene hits in any *T. erythraeum* MAGs in our dataset or from NCBI [13]. Finally, the observation that the *T. erythraeum* IMS101 genome is closed [12] and completely missing the CRISPR-Cas system, supports its absence from *T. erythraeum* in general.

As the *cas10* gene is diagnostic for the Type III-B CRISPR predicted to be encoded by *T. thiebautii* and can show significant sequence variation [68], we performed phylogenetic analyses of Cas10 protein sequences to explore the origins of this system in the lineage. The Cas10 maximum likelihood phylogeny shown in Fig. 4B suggests two-to-three Type III-B systems in *T. thiebautii*. Additionally, this tree indicates that these systems are likely ancestral because the phylogeny of each of the three distinct sequence clusters is roughly congruent with the phylogenomic signal shown in Fig. 4A. However, careful comparison of both trees shows that all three Type III-B Cas10 protein clusters are not conserved in every *T. thiebautii* assembly. BLASTN searches confirmed that the missing *cas10* genes were present at contig breaks in our *T*. *thiebautii* MAGs (i.e., St18_bin1, St19_bin1, and St16_bin2_tuft) corresponding to clusters 1 & 2 in Fig. 4B. The most straightforward interpretation of these data is that most *T. thiebautii* assemblies do not have cluster 3, and perhaps it is currently disappearing from the *Trichodesmium* pangenome. Fittingly, cluster 3 is undetectable from our best-assembled MAG, *T. thiebautii* H94 isolate genome (566 contigs). Thus, while there is variation in III-B CRISPR-Cas system copy number in *T. thiebautii*, its loss in *T. erythraeum* is a phylogenetically constrained defining difference between these major *Trichodesmium* clades.

Generally speaking, CRISPR-Cas systems protect the cell from mobile genetic elements (MGEs; phage and mobile plasmids) via a sequence-based, targeted genome degradation [69, 70]. Many different CRISPR-Cas systems that vary in gene content and recognition molecule (RNA vs. DNA) have been described [20]. That said, while all CRISPR-Cas variants appear to provide memory-driven immunity against MGEs, the Type III-B subtype, predicted in numerically abundant *T. thiebautii* clades, requires active RNA transcription for function, can use other CRISPR arrays in addition to its own, and provides better protection against phage protospacer mutagenic evasion [71].

Mechanistically, Type III-B CRISPR-Cas systems operate in three steps (1) adaptation: recognition and incorporation of transcribed 30–50 bp protospacers (i.e., DNA or RNA sequences of invading MGEs; typically mediated by Cas1 or Cas1-reverse transcriptase (RT) fusion proteins, respectively [72, 73]) into CRISPR arrays as spacer sequence DNA “memories” of past attacks, (2) expression: spacer RNAs are expressed as precursor CRISPR RNA (crRNA), and (3) interference: sequence-specific crRNAs guides interfere with invading phage or plasmids by the action of the Cas10 protein [68, 69]. The absence of a Cas1-RT fusion protein in *T. thiebautii* suggests that the primary adaptation targets for this system are transcribed DNA MGEs. In contrast, an HD superfamily nuclease domain in *T. thiebautii* Cas10 proteins indicates that the interference step is likely cleaving both RNA and transcribed DNA [68, 72, 74]. Importantly, these DNA spacer sequences also provide ‘fingerprints’ of past MGE attacks that link phage/plasmid sequences with the CRISPR-Cas system containing host (e.g. [75–77]).

### Predicted phage genome fragments assembled from TriCoLim 100% match *T. thiebautii* CRISPR spacers

We next sought to identify predicted MGEs from colony assemblies and determine if they matched *T. thiebautii* spacer sequences. While metaplasmidSPAdes identified several putative plasmids in enrichment and field samples (data not shown), none matched any *T. thiebautii* spacers. We also could not detect phage particles/genomes from the enrichment MAGs; however, the TriCoLim assemblies revealed 1000 s of putative phage genome fragments with contigs sizes ranging from 1000 s to >100kbps (data not shown).

Next, we asked whether these phage genomes sequences matched the *T. thiebautii* spacers. This effort identified seven 100% ID hits and 29 more with ANI > 90% covering ≥93% of the spacer (Supplementary Table 4). We conservatively picked the latter ID and coverage level because Type III-B crisper systems can function with mismatches, a feature that requires phage to delete ‘whole’ spacer-protospacer targets from their genomes to escape degradation [78, 79]. Unfortunately, these spacer-matching putative phage DNA fragments only ranged from 1755 to 5628 bps and were thus too small to identify the phage. All spacer-matching contigs were categorized as virstorter2 category 2 (i.e., likely phage DNA) and contained many predicted hypothetical viral genes, while one also is expected to encode a transposase (Supplementary Table 5). None of the fragments drew hits from known phage sequences in Genbank or IMG or any other gene. It is noteworthy that many of these putative phage genome fragments were detected multiple times from independently assembled TriCoLim stations (Supplementary Table 5; fastANI groupings), suggesting that some consistent phage particles were present across the transect. As past research shows that high phage relatedness selects for CRISPR-Cas systems [69, 80, 81], these data suggest that the *T. thiebautii* CRISPR-Cas system is defending against a relatively conserved phage group.

### *T. thiebautii* CRISPR-Cas systems are expressed in situ

**Fig. 5:**
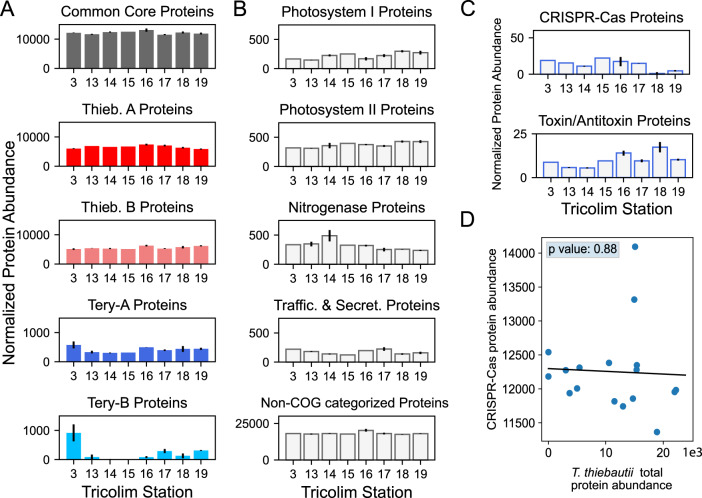
*Trichodesmium* protein abundances across the TriCoLim transect. **A** Protein abundance data sorted into *Trichodesmium* phylogenetic groups. Proteins were normalized across each sample, then sorted into the respective phylogenetic group and summed. Error bars indicate the standard deviation of the averaged biological replicates. Quantitation is displayed as normalized spectral counts (see “Methods”). Core and *T. thiebautii* proteins are much more abundant than those derived from *T. erythraeum*. **B** Protein abundance data sorted by biological function. Again, proteins were normalized across each sample, then sorted by COG function and summed. Selected, highly abundant functions are shown. **C** Summed normalized protein data for CRISPR-Cas and toxin/antitoxin proteins across the TriCoLim transect. **D** Nonsignificant correlation of CRISPR-Cas protein abundances versus *T. thiebautii* total protein abundance (*p* = 0.88).

Identifying conserved *Trichodesmium* spacers and putative phage genome fragments suggests that the CRISPR-Cas systems are active in the field. To verify, we screened our TriCoLim *Trichodesmium* colony metaproteomics dataset for in situ Cas protein expression (protein identifications are provided as Supplementary Tables 6–8). In this re-analysis, we identified 3498 proteins and 68058 peptides. After binning the detected proteins by clade, *T. thiebautii* proteins were >10x more abundant across the transect than either *T. erythraeum* clades (Fig. 5A—Red, Pink, Blue, and Cyan filled colored bars, respectively). This protein detection dataset agrees well with our metagenomic read mapping in Fig. 3B, where *T. thiebautii* consistently dominated the sequencing reads. Major metabolic proteins such as the photosystems and nitrogenase were detected in high levels across the transect (Fig. 5B) and originated mainly from *T. thiebautii* proteins. Non-COG categorized proteins were ×50 more abundant across the transect than even these core metabolic functions, consistent with this being one of the most enriched categories in the *T. thiebautii* assemblies. Additionally, the in situ expression of these non-categorized proteins suggests that they are required for environmental growth and highlights the importance of characterizing them further.

Proteins involved in cellular defense, including toxin/antitoxin proteins (i.e., the toxin components of RelE and MazEF and the antitoxin component of ParD) and the CRISPR-Cas system, were identified in across the transect (Fig. 5C). The CRISPR-Cas proteins did not correlate with total *T. thiebautii* protein abundance, suggesting that the former are not constitutively expressed (i.e., as a function of biomass) and are instead under some regulatory control. The CRISPR proteins identified included Cas10 and Cas7, and their phylogenetic assignment at the peptide level corresponded to *T. thiebautii* species and were assigned to Cas10 clusters 1 & 2 from Fig. 4B. Specifically, we identified peptides that were identical to those in *T. thiebautii* MAGS H94, St18_bin1, St16_bin2, and MAG *T. thiebautii* Indian Ocean, indicating that these species were contributing to CRISPR-Cas protein production in situ (Supp. Table 8). These data also show that Thieb-B clade members are present and active in the Atlantic Ocean.

Research shows that CRISPR-Cas adaptation (i.e., protospacer incorporation into spacer arrays) requires Cas1 or Cas2 to respond to new MGE threats [20, 73]. Thus the absence of these proteins in our metaproteome could suggest that the *T. thiebautii* CRISPR-Cas system is not actively adapting to new phage and is perhaps performing alternative functions in the cell independent of viral immunity [82, 83]. Three observations argue against this supposition. (1) self-targeting spacers (i.e., matching alternative sites in the MAG) were not identified, suggesting that interference-based gene regulation is not occurring (e.g. [82–84]). (2) most of the spacers detected in each MAG are distinct from those in other MAGs, suggesting that “rapid” adaptation occurs in *T. thiebautii* [75, 85], (3) read recruiting from Pacific Ocean *Trichodesmium* community data collected by others [86] shows that all annotated *T. thiebautii* H94 *cas* genes are expressed (including *cas1* and *cas2*), and they appear to have diel periodicity (Supplementary Fig, 2; gene targets and results in Supp. Tables 9–12). Thus, while we cannot exclude alternative CRISPR functions in *T. thiebautii*, our data strongly suggest CRISPR-Cas mediated phage immunity is commonplace in the clade.

## Conclusion

We show that the conserved maintenance of a functional CRISPR-Cas system in *T. thiebautii* is a defining speciation difference between the major clades of *Trichodesmium*. This conservation combined with singleton gene enrichment suggests that immunity allows the recipients of transduced genes to survive and thereby increase their genetic diversity (as noted in other systems [87, 88]). More research is needed to determine if CRISPR-Cas immunity conservation in *T. thiebautii* is the cause or the result of their numerical dominance over *T. erythraeum*. Because we are only just beginning to address how MGE selection maintains CRISPR-Cas systems in global populations [80], our findings also highlight the importance of future *Trichodesmium* virome studies. For example, will *T. erythraeum* be a climate change winner as predicted [15, 89] or will increased phage infectivity reduce their future expansion?

## Supplementary Information

Supplemental Figure

Supplemental Table

Spplementary information

## Data Availability

Our sequences files are accessible from the National Center for Biotechnology Information (NCBI; BioProject PRJNA828267), Sequence Read Archive accession numbers SRR19658988 through SRR19658999. MAG Assemblies are available at NCBI Assembly page with accessions SAMN29146503 through SAMN29146503. The mass spectrometry proteomics data were originally deposited in the ProteomeXchange Consortium via the PRIDE partner repository with the identifier PXD016225 and can be accessed at 10.6019/PXD016225 [90, 91]. The data is also available at BCO-DMO (https://www.bco-dmo.org/dataset/787078).
